# *Vital Signs: *Health Worker–Perceived Working Conditions and Symptoms of Poor Mental Health — Quality of Worklife Survey, United States, 2018–2022

**DOI:** 10.15585/mmwr.mm7244e1

**Published:** 2023-11-03

**Authors:** Jeannie A. S. Nigam, R. Michael Barker, Thomas R. Cunningham, Naomi G. Swanson, L. Casey Chosewood

**Affiliations:** ^1^Division of Science Integration, National Institute for Occupational Safety and Health, CDC; ^2^Office of the Director, National Institute for Occupational Safety and Health, CDC.

**Summary**

**What is already known about this topic?**

The longstanding health worker burnout crisis preceded the COVID-19 pandemic, which began in 2020.

**What is added by this report?**

Health worker respondents to the General Social Survey Quality of Worklife Module reported more days of poor mental health and were more likely to report burnout in 2022 than in 2018. Positive working conditions, such as trust in management and supervisor help, were associated with lower odds of poor mental health symptoms and burnout.

**What are the implications for public health practice?**

Health workers continued to face a mental health crisis in 2022. The National Institute for Occupational Safety and Health has developed a campaign, Impact Wellbeing, to provide employers of health workers with resources to modify working conditions and improve worker mental health, thereby supporting the nation’s health system.

## Abstract

**Introduction:** Health workers faced overwhelming demands and experienced crisis levels of burnout before the COVID-19 pandemic; the pandemic presented unique challenges that further impaired their mental health.

**Methods:** Data from the General Social Survey Quality of Worklife Module were analyzed to compare self-reported mental health symptoms among U.S. adult workers from 2018 (1,443 respondents, including 226 health workers) and 2022 (1,952, including 325 health workers). Logistic regression was used to examine associations between health workers’ reported perceptions of working conditions and anxiety, depression, and burnout.

**Results:** From 2018 to 2022, health workers reported an increase of 1.2 days of poor mental health during the previous 30 days (from 3.3 days to 4.5 days); the percentage who reported feeling burnout very often (11.6% to 19.0%) increased. In 2022, health workers experienced a decrease in odds of burnout if they trusted management (odds ratio [OR] = 0.40), had supervisor help (OR = 0.26), had enough time to complete work (OR = 0.33), and felt that their workplace supported productivity (OR = 0.38), compared with those who did not. Harassment at work was associated with increased odds of anxiety (OR = 5.01), depression (OR = 3.38), and burnout (OR = 5.83).

**Conclusions and implications for public health practice:** Health workers continued to face a mental health crisis in 2022. Positive working conditions were associated with less burnout and better mental health. CDC’s National Institute for Occupational Safety and Health has developed a national campaign, Impact Wellbeing, to provide employers of health workers with resources to improve the mental health of these workers.

## Introduction

Work in health occupations* (which include clinicians as well as those in mental health, public health, long-term care, and other support roles) is stressful owing to demanding working conditions^†^ including taxing work; exposure to infectious diseases; long hours; and challenging interactions with coworkers, patients, and their families. Chronic exposure to stressful working conditions, including not participating in decision-making (*1*) and lack of supportive supervision (*2*), can lead to mental strain, and during the COVID-19 pandemic, contributed to health worker turnover (*3*,*4*). Depressive disorders are a leading cause of disability (*5*), and for workers, are associated with higher rates of absenteeism and presenteeism (working when physically ill) (*6*). In 2021, one in four U.S. essential workers (including health workers) had received a mental disorder diagnosis since the pandemic onset (*7*).

U.S. health workers experienced a 249% increase in rates of work-related injury and illness between 2019 and 2020.^§^ The pandemic intensified existing risks and workloads because of staff member shortages, high patient loads, supply shortages, fatigue, and grief, exacerbating preexisting crisis levels of burnout (e.g., feeling emotionally exhausted and detached and experiencing a low sense of personal accomplishment at work) (*8*). Health workers experienced increased harassment (i.e., threats, bullying, verbal abuse, or other actions from patients and coworkers that create a hostile work environment) and violence (*9*), which can increase the risk for symptoms of depression, anxiety, posttraumatic stress, and suicidal ideation (*10*). The purpose of this analysis was to ascertain whether U.S. health workers experienced more mental health declines than did other workers during the COVID-19 pandemic.

This report describes and compares self-reported well-being and working conditions for health workers, other essential workers, and all other workers in 2018 and 2022 using cross-sectional data from the Quality of Worklife (QWL) module of the nationally representative General Social Survey (GSS).^¶^ To identify potential prevention strategies, working conditions associated with frequency of symptoms of anxiety, depression, and burnout for health workers in 2022 were examined.

## Methods

The QWL module contains questions on working and mental health conditions and is administered to respondents aged ≥18 years within GSS who report having been employed during the preceding 2 weeks. Items from the GSS/QWL module** for 2018 (17 items, administered via personal interview) and 2022 (25 items, including eight new items, administered via personal interview, telephone interview, or web-based questionnaire) were analyzed to examine working conditions and related outcomes before and after the onset of the COVID-19 pandemic and across worker groups.^††^ The total sample comprised 3,395 respondents. In 2018, respondents included 1,443 workers (226 health workers, 379 other essential workers, and 838 other workers [“all other workers”]). In 2022, respondents included 1,952 workers (325 health workers, 467 other essential workers, and 1,160 other workers). Response rates for GSS were 59.5% in 2018 and 50.5% in 2022.

Perceptions of working conditions were measured using five single ordinal items that asked respondents whether 1) they trust management, 2) they were harassed at work, 3) there was enough time to accomplish work, 4) working conditions supported productivity, and 5) supervisors were helpful. Two single ordinal items asked how often there were enough persons or staff members to complete work and whether the respondent participated in decision-making. A composite measure of psychosocial safety climate (*11*), added to the QWL in 2022, was also included.^§§^ Worker-reported well-being outcomes including general happiness, frequency of sleep problems, days of poor mental health during the previous 30 days (e.g., stress, feeling depressed, and problems with emotions), and turnover intention (intent to find a new job in the next year), were measured by single ordinal items. Presenteeism, added to the QWL in 2022, was also measured by a single ordinal item.

To determine which working conditions were associated with adverse mental health outcomes among health workers in 2022, comparisons of prevalences of self-reported burnout during the previous month, and anxiety and depression during the previous 2 weeks were made across different working conditions. Burnout was measured with a single item about feeling “used up.” Anxiety and depression were each measured by two items added to the QWL in 2022 from the four-item Patient Health Questionnaire (PHQ-4), a screening tool for anxiety and depression (*12*); scores (range = 0–3) for the two corresponding items were summed (range = 0–6) then dichotomized such that scores of ≥1 indicated the presence of at least one symptom for several days during the previous 2 weeks.

Differences between worker groups and survey year (i.e., a three by two interaction) for the selected outcomes were analyzed using generalized linear modeling (GLM). Weighted percentages of responses and Wald 95% CIs were estimated from these models. The statistical significance of the main effect of year, worker group, and the interaction was determined by evaluating the improvement in model fit when the effect was added to the model. Fit comparisons were made with a likelihood ratio test; Wald chi-square tests with p<0.05 indicated better model fit. CIs were inspected when the interaction was significant; nonoverlapping CIs indicated statistically significant differences at p<0.05. All differences reported were statistically significant. Binary logistic regression, ordinal logistic regression, and zero-inflated Poisson regression were used for dichotomous outcomes, ordinal outcomes, and count outcomes with zero-inflation, respectively. Separate bivariate logistic regressions were conducted (using GLM with a logit-link and a binomial distribution) to evaluate the association between working conditions and anxiety symptoms, depression symptoms, and burnout in the health worker group. As before, the statistical significance of the working condition variable was determined by comparison to a null model via likelihood ratio test. Odds ratios, Wald 95% CIs, and weighted percentages of responses were estimated from these models. All statistical analyses were conducted in R (version 4.2.2; The R Foundation) using the svyVGAM package (version 1.2; Thomas Lumley [developer]) to account for the complex sampling design and weighting of GSS. This activity was reviewed by CDC, deemed not research, and was conducted consistent with applicable federal law and CDC policy.^¶¶^

## Results

Distribution of survey respondents by age and gender varied by worker group. In both years, health workers and other essential workers were more likely to be women than were respondents in the other worker group. The proportion of persons earning <$35,000 per year decreased in 2022 from 2018 for each worker group (Table 1).

**TABLE 1:** Demographic characteristics* of health workers, other essential workers,^†^ and all other workers — General Social Survey Quality of Worklife Module, United States, 2018 and 2022

Characteristic	% (95% CI)					
	Health workers		Other essential workers		All other workers	
	2018 n = 226	2022 n = 325	2018 n = 379	2022 n = 467	2018 n = 838	2022 n = 1160
**Age group, yrs**						
<30	27.4 (19.7–36.7)	26.3 (18.6–35.9)	16.9 (11.8–23.6)	22.1 (17.5–27.5)	29.2 (25.4–33.3)	25.9 (22.0–30.2)
30–39	15.9 (10.4–23.4)	21.2 (16.7–26.6)	21.5 (17.3–26.3)	14.8 (10.9–19.9)	18.2 (15.4–21.4)	20.8 (17.9–24.0)
40–49	20.8 (14.7–28.6)	21.5 (13.7–32.2)	25.3 (19.8–31.8)	24.9 (19.8–30.7)	17.6 (14.2–21.5)	20.1 (17.2–23.5)
50–59	21.9 (15.8–29.6)	21.3 (14.8–29.7)	22.1 (17.3–27.8)	18.4 (14.6–23.0)	22.0 (19.1–25.2)	19.6 (16.2–23.5)
≥60	14.0 (9.8–19.7)	9.6 (5.8–15.5)	14.2 (11.0–18.1)	19.8 (13.9–27.5)	13.0 (10.5–16.0)	13.6 (10.6–17.2)
**Gender (women)**	75.8 (65.9–83.5)	71.4 (63.7–78.1)	49.7 (43.3–56.2)	51.2 (45.4–57.1)	40.4 (35.7–45.2)	40.9 (36.5–45.5)
**Race and ethnicity**						
A/PI, NH	6.7 (2.7–15.6)	9.4 (4.2–19.6)	3.3 (1.1–9.4)	4.9 (2.8–8.4)	4.9 (3.4–7.0)	3.6 (2.2–5.8)
AI/AN, NH	0 (0–0)^§^	0.8 (0.3–2.1)	0.7 (0.2–2.2)	0.2 (0.1–0.5)	0.3 (0.1–0.8)	0.2 (0.1–0.7)
Black or African American, NH	11.2 (7.1–17.1)	17.7 (11.9–25.5)	12.1 (8.6–16.9)	8.6 (6.1–12.0)	9.4 (7.1–12.4)	10.4 (7.5–14.2)
White, NH	66.3 (57.0–74.6)	59.0 (49.9–67.5)	58.3 (51.4–64.9)	70.3 (64.3–75.6)	59.2 (54.7–63.5)	63.0 (57.6–68.1)
Hispanic or Latino	13.1 (8.8–19.0)	10.1 (7.3–13.9)	18.8 (13.8–25.1)	14.1 (9.5–20.5)	17.9 (14.4–22.2)	17.1 (13.6–21.3)
Multiple races, NH	2.5 (1.1–5.5)	2.6 (1.0–7.0)	6.3 (4.1–9.6)	1.9 (0.9–4.1)	7.3 (5.5–9.7)	5.3 (3.4–8.0)
Other race, NH	0.2 (0–1.4)^§^	0.4 (0.1–1.6)	0.5 (0.1–1.7)	0 (0–0)^§^	0.9 (0.3–2.7)	0.3 (0.1–0.8)
**Education**						
No high school diploma	2.4 (0.8–6.9)	6.1 (2.5–13.9)	9.0 (5.9–13.3)	3.6 (1.6–8.2)	10.9 (7.6–15.3)	10.8 (8.1–14.2)
High school diploma	41.2 (32.8–50.2)	32.3 (24.3–41.4)	41.9 (35.8–48.2)	38.8 (31.8–46.3)	52.1 (46.9–57.2)	52.9 (48.0–57.6)
Associate college or junior college degree	13.6 (9.0–20.1)	15.2 (10.3–21.9)	8.2 (5.8–11.3)	9.3 (6.2–13.6)	7.0 (5.1–9.7)	8.1 (5.7–11.4)
Bachelor's degree	22.3 (16.3–29.7)	30.1 (21.9–39.7)	24.5 (19.4–30.5)	27.3 (21.7–33.6)	20.3 (16.9–24.2)	19.0 (15.9–22.5)
Graduate degree	20.6 (13.2–30.6)	16.4 (11.7–22.4)	16.5 (11.4–23.4)	21.1 (15.8–27.5)	9.7 (7.3–12.9)	9.3 (7.1–12.1)
**Income**						
<$35,000	40.4 (31.7–49.7)	33.9 (25.0–44.2)	43.8 (36.9–50.8)	28.1 (22.9–34.0)	46.0 (41.9–50.2)	39.0 (33.6–44.6)
$35,000–$74,999	39.0 (28.8–50.4)	38.4 (30.1–47.4)	35.1 (29.4–41.3)	42.6 (36.3–49.2)	29.7 (25.8–33.8)	28.6 (24.3–33.3)
$75,000–$149,999	16.1 (10.2–24.7)	20.9 (14.8–28.6)	18.6 (14.0–24.2)	22.3 (16.7–29.0)	17.4 (14.0–21.4)	22.5 (18.7–26.8)
≥$150,000	4.5 (2.2–8.9)	6.8 (3.5–13.1)	2.6 (1.0–6.3)	7.0 (3.9–12.2)	6.9 (4.5–10.3)	10.0 (6.8–14.5)

**Abbreviations**: AI/AN = American Indian or Alaska Native; A/PI = Asian or Pacific Islander; NH = non-Hispanic.

* All analyses used survey weights provided by the General Social Survey.

^†^ Frontline, nonhealth workers.

^§^ Value displayed as 0 due to rounding.

The overall number of poor mental health days in the previous 30 days in 2022 was similar across all three groups of workers (4.1–4.5 days)*** (Table 2). Health workers, however, reported a significant increase in poor mental health days in the previous 30 days from 2018 (3.3 days) to 2022 (4.5 days). During this period, the percentage of health workers who reported feeling burnout very often increased from 11.6% to 19.0%. Overall, 45.6% of health workers reported feeling burnout often or very often in 2022. The percentage of health workers who reported feeling very happy did not change significantly from 2018 to 2022, but rates of feeling very happy did decline among other essential workers and all other workers (from 33.9% to 20.5% and from 33.6% to 26.3%, respectively).

**TABLE 2:** Mental health, well-being, and working conditions* of health workers, other essential workers,**^†^** and all other workers — General Social Survey Quality of Worklife Module, United States, 2018 and 2022

Variable	Estimate, % (95% CI)					
	Health workers		Other essential workers		All other workers	
	2018 n = 226	2022 n = 325	2018 n = 379	2022 n = 467	2018 n = 838	2022 n = 1,160
**General happiness^§,¶^**						
Not too happy**	12.8 (12.6–13.1)	14.1 (13.9–14.3)	11.9 (11.7–12.1)	21.2 (21.0–21.4)	12.1 (11.9–12.2)	16.3 (16.2–16.4)
Pretty happy	55.2 (52.3–57.6)	56.2 (53.7–58.4)	54.2 (52.2–55.9)	58.3 (55.5–60.9)	54.4 (53.0–55.6)	57.5 (56.0–58.8)
Very happy	32.0 (27.3–37.1)	29.7 (26.0–33.7)	33.9 (30.3–37.7)	20.5 (18.0–23.2)	33.6 (31.2–36.0)	26.3 (24.4–28.1)
**Sleep problems^††,§§^**						
Never**	14.4 (14.2–14.7)	11.0 (10.8–11.2)	13.6 (13.4–13.7)	12.0 (11.8–12.1)	14.4 (14.3–14.5)	12.3 (12.2–12.4)
Rarely	28.7 (25.7–31.2)	24.7 (22.8–26.4)	27.8 (25.7–29.7)	26.0 (24.2–27.5)	28.7 (27.1–30.1)	26.4 (25.2–27.5)
Sometimes	35.8 (30.5–40.9)	37.6 (33.5–41.5)	36.3 (32.5–40.1)	37.2 (33.7–40.6)	35.8 (33.3–38.4)	37.0 (34.9–39.1)
Often	21.1 (17.7–24.9)	26.7 (23.4–30.3)	22.3 (19.6–25.2)	24.9 (22.2–27.8)	21.1 (19.3–23.0)	24.3 (22.6–26.1)
**Mean days of poor mental health (previous 30 days)^¶¶,¶^**	3.3 (3.0–3.6)	4.5 (4.2–4.9)	3.7 (3.4–3.9)	4.1 (3.8–4.3)	3.8 (3.6–4.0)	4.3 (4.0–4.5)
Anxiety symptoms (Yes)***	NA	57.0 (52.3–61.6)	NA	53.1 (49.1–57.1)	NA	51.8 (49.5–54.1)
**Depression symptoms (Yes)^†††^**	NA	34.4 (30.1–39.0)	NA	38.5 (34.7–42.4)	NA	41.8 (39.5–44.1)
**Burnout^§§§,¶^**						
Never**	10.7 (10.4–10.9)	6.3 (6.1–6.4)	8.1 (7.9–8.2)	7.8 (7.7–8.0)	9.6 (9.5–9.7)	8.7 (8.6–8.8)
Rarely	21.4 (19.1–23.4)	14.7 (13.4–15.7)	17.7 (16.3–19.0)	17.4 (16.1–18.5)	20.0 (18.8–21.1)	18.7 (17.7–19.6)
Sometimes	36.0 (31.2–40.5)	33.5 (30.5–36.3)	35.3 (32.1–38.2)	35.1 (32.2–37.8)	35.9 (33.7–38.1)	35.6 (33.8–37.4)
Often	20.3 (15.9–25.1)	26.6 (22.4–30.8)	23.8 (20.1–27.6)	24.1 (20.7–27.6)	21.6 (19.3–24.0)	22.9 (20.9–25.0)
Very often	11.6 (9.5–14.0)	19.0 (16.4–21.9)	15.1 (13.1–17.4)	15.5 (13.6–17.7)	12.8 (11.5–14.2)	14.1 (12.9–15.5)
Presenteeism (Yes)^¶¶¶,^****	NA	27.9 (23.8–32.3)	NA	43.2 (39.2–47.2)	NA	37.4 (35.1–39.6)
**Turnover intention^††††,¶^**						
Not at all likely**	66.6 (66.3–66.8)	55.9 (55.7–56.1)	60.1 (60.0–60.3)	67.8 (67.6–68.0)	52.2 (52.1–52.3)	61.1 (61.0–61.2)
Somewhat likely	22.3 (17.1–28.0)	27.7 (23.2–32.2)	25.7 (21.6–29.8)	21.6 (18.0–25.4)	29.2 (26.5–31.9)	25.2 (23.0–27.5)
Very likely	11.1 (8.9–13.9)	16.5 (14.0–19.3)	14.2 (12.1–16.5)	10.6 (9.0–12.4)	18.6 (16.9–20.5)	13.7 (12.5–15.1)
**Harassed at work (Yes)^§§§§,¶^**	6.4 (4.0–10.0)	13.4 (10.5–16.9)	7.9 (5.8–10.5)	10.8 (8.6–13.6)	7.0 (5.7–8.5)	6.6 (5.5–7.9)
**Psychosocial safety climate^¶¶¶¶^**						
Poor**	NA	9.0 (8.8–9.2)	NA	12.0 (11.8–12.2)	NA	10.9 (10.8–11.0)
Moderate	NA	18.1 (16.0–19.8)	NA	21.9 (19.7–23.7)	NA	20.5 (19.2–21.8)
Good	NA	72.9 (68.5–76.9)	NA	66.1 (62.3–69.8)	NA	68.6 (66.4–70.7)
Supervisor help*****^,^****						
Not at all true**	3.3 (3.0–3.5)	4.3 (4.1–4.5)	5.8 (5.6–5.9)	5.2 (5.0–5.3)	4.4 (4.3–4.5)	4.2 (4.1–4.3)
Not too true	6.9 (5.9–7.7)	8.8 (7.8–9.6)	11.2 (10.0–12.2)	10.2 (9.2–11.1)	8.9 (8.2–9.6)	8.6 (7.9–9.2)
Somewhat true	31.1 (28.6–33.1)	35.2 (32.8–37.2)	38.9 (36.3–41.2)	37.5 (35.3–39.5)	35.5 (33.9–36.9)	34.8 (33.5–36.0)
Very true	58.7 (53.0–64.3)	51.8 (47.2–56.3)	44.2 (40.2–48.3)	47.1 (43.3–50.9)	51.2 (48.4–53.9)	52.5 (50.2–54.7)
**Trust in management^†††††,¶^**						
Strongly disagree**	3.7 (3.5–4.0)	5.3 (5.1–5.5)	4.5 (4.3–4.7)	5.7 (5.5–5.9)	3.9 (3.7–4.0)	3.3 (3.2–3.5)
Disagree	12.4 (11.4–13.3)	16.5 (15.3–17.5)	14.5 (13.5–15.3)	17.4 (16.2–18.4)	12.8 (12.1–13.4)	11.4 (10.8–11.9)
Agree	55.1 (51.7–58.0)	56.4 (53.0–59.4)	56.1 (53.3–58.6)	56.3 (53.4–59.0)	55.4 (53.6–57.0)	54.2 (52.8–55.5)
Strongly agree	28.8 (24.3–33.7)	21.8 (18.7–25.1)	24.9 (21.9–28.3)	20.6 (18.1–23.3)	27.9 (25.7–30.2)	31.1 (29.1–33.1)
Time to get job done^§§§§§,^****						
Not at all true**	3.2 (2.9–3.4)	3.2 (3.0–3.4)	5.4 (5.2–5.5)	5.8 (5.6–6.0)	3.4 (3.2–3.5)	3.6 (3.5–3.8)
Not too true	8.8 (7.9–9.5)	8.9 (8.1–9.5)	13.8 (12.6–14.7)	14.6 (13.5–15.6)	9.4 (8.8–9.9)	10.0 (9.3–10.5)
Somewhat true	39.9 (37.2–42.1)	40.0 (37.8–41.9)	46.0 (43.3–48.5)	46.6 (43.9–49.0)	40.9 (39.4–42.2)	41.9 (40.6–43.1)
Very true	48.2 (42.6–53.8)	48.0 (43.5–52.5)	34.8 (31.2–38.7)	33.0 (29.7–36.4)	46.4 (43.7–49.1)	44.5 (42.3–46.8)
**Takes part in decisions^¶¶¶¶¶,§§^**						
Never**	8.6 (8.4–8.9)	7.7 (7.5–7.8)	11.2 (11.1–11.4)	8.1 (7.9–8.2)	8.5 (8.4–8.6)	7.8 (7.7–7.9)
Rarely	16.2 (14.1–17.8)	14.8 (13.3–16.0)	19.3 (17.5–21.0)	15.4 (14.0–16.5)	16.0 (14.9–17.0)	15.0 (14.1–15.8)
Sometimes	37.8 (33.5–41.7)	37.1 (33.8–40.1)	38.6 (35.0–41.9)	37.4 (34.6–40.1)	37.8 (35.7–39.8)	37.2 (35.5–38.9)
Often	37.4 (32.4–42.6)	40.5 (36.3–44.8)	30.9 (27.6–34.4)	39.1 (35.7–42.7)	37.7 (35.3–40.3)	39.9 (37.8–42.1)
Conditions support productivity******^,¶^						
Strongly disagree**	0.9 (0.7–1.2)	2.1 (1.9–2.3)	1.7 (1.5–2.0)	2.8 (2.5–3.0)	1.1 (1.0–1.3)	1.1 (1.0–1.3)
Disagree	8.2 (7.8–8.4)	16.4 (15.7–16.9)	14.0 (13.4–14.4)	20.1 (19.3–20.8)	9.7 (9.4–9.9)	9.6 (9.3–9.8)
Agree	60.5 (58.2–62.3)	65.3 (62.2–68.1)	65.3 (62.7–67.6)	64.3 (61.2–67.0)	62.7 (61.3–63.9)	62.6 (61.4–63.6)
Strongly agree	30.4 (25.6–35.6)	16.2 (13.6–19.1)	19.0 (16.4–22.0)	12.8 (11.0–14.9)	26.5 (24.3–28.8)	26.7 (24.8–28.7)
**Not enough staff members^††††††,¶^**						
Never**	12.8 (12.6–13.1)	9.8 (9.6–9.9)	10.6 (10.4–10.8)	7.7 (7.6–7.9)	14.8 (14.7–15.0)	15.0 (14.9–15.1)
Rarely	25.1 (22.4–27.4)	21.2 (19.4–22.8)	22.4 (20.6–23.9)	18.0 (16.8–19.1)	27.1 (25.6–28.6)	27.3 (25.9–28.6)
Sometimes	36.3 (31.1–41.3)	37.0 (33.1–40.6)	36.9 (33.3–40.4)	36.3 (33.4–39.1)	35.4 (32.8–37.9)	35.3 (33.1–37.4)
Often	25.7 (21.8–30.1)	32.0 (28.4–36.0)	30.1 (26.8–33.6)	37.9 (34.5–41.4)	22.6 (20.8–24.6)	22.4 (20.8–24.1)

**Abbreviations**: GSS = General Social Survey; NA = not available.

* All analyses used survey weights provided by GSS.

^†^ Frontline, nonhealth workers.

^§^ “Taken all together, how would you say things are these days, would you say that you are very happy, pretty happy, or not too happy?” (GSS variable name: happy).

^¶^ Significant interaction between worker group and year per likelihood ratio test (p<0.05).

** CIs for the lowest level of ordinal scales were calculated using the pooled SE for the other categories in the scale.

^††^ “During the past 12 months, how often have you had trouble going to sleep or staying asleep?” (GSS variable name: slpprblm).

^§§^ Significant main effect for year per likelihood ratio test (p<0.05).

^¶¶^ “Now thinking about your mental health, which includes stress, depression, and problems with emotions, for how many days during the past 30 days was your mental health not good?” Numeric responses range = 0–30 (GSS variable name: mntlhlth).

*** Composite of GSS variables feelnerv (“Over the last 2 weeks, how often have you been bothered by the following problems: feeling nervous, anxious, or on edge”) and worry (“Over the last 2 weeks, how often have you been bothered by the following problems: not being able to stop or control worrying”). Response options: not at all (0), several days (1), more than half the days (2), nearly every day (3). Items were summed and scores of ≥1 were coded as “Yes” for anxiety symptoms.

^†††^ Composite of GSS variables feeldown (“Over the last 2 weeks, how often have you been bothered by the following problems: feeling down, depressed, or hopeless”) and nointerest (“Over the last 2 weeks, how often have you been bothered by the following problems: little interest or pleasure in doing things”). Response options: not at all (0), several days (1), more than half the days (2), nearly every day (3). Items were summed and scores of ≥1 were coded as “Yes” for depression symptoms.

^§§§^ “How often during the past month have you felt used up at the end of the day?” (GSS variable name: usedup).

^¶¶¶^ New item for 2022. “During the past 3 months, how many days did you work while physically ill?” Scores of ≥1 were recoded as “Yes” for presenteeism (GSS variable name: worksick).

**** Significant main effect for worker group per likelihood ratio test (p<0.05).

^††††^ “Taking everything into consideration, how likely is it you will make a genuine effort to find a new job with another employer within the next year?” (GSS variable name: trynewjb).

^§§§§^ “In the last 12 months, were you threatened or harassed in any other way by anyone while you were on the job?” (GSS variable name: wkharoth).

^¶¶¶¶^ New items for 2022. Composite of GSS variables psysamephys (“Senior management considers psychological health to be as important as productivity”), strmgtsup (“Senior management show support for stress prevention through involvement and commitment”), and allorglevel (“In my organization, the prevention of stress involves all levels of the organization”). Response options: strongly disagree (1), disagree (2), neither agree nor disagree (3), agree (4), and strongly agree (5). Items were summed and scores <6 were coded “poor,” 6–8 were coded “moderate,” and ≥9 were coded “good.”

***** “My supervisor is helpful to me in getting the job done” (GSS variable name: suphelp).

^†††††^ “I trust the management at the place where I work” (GSS variable name: trustman).

^§§§§§^ “I have enough time to get the job done” (GSS variable name: wrktime).

^¶¶¶¶¶^ “In your job, how often do you take part with others in making decisions that affect you?” (GSS variable name: wkdecide).

****** “Conditions on my job allow me to be about as productive as I could be” (GSS variable name: prodctiv).

^††††††^ “How often are there not enough people or staff to get all the work done?” (GSS variable name: toofewwk).

From 2018 to 2022, the percentage of health workers who reported being very likely to look for a new job with another employer increased from 11.1% to 16.5%; overall, 44.2% of health workers reported being somewhat likely or very likely to look for a new job in 2022. In contrast, among all other workers, turnover intention declined from 18.6% to 13.7% during this period. Health workers’ reports of being harassed at work more than doubled, from 6.4% in 2018 to 13.4% in 2022. The rates of trusting management decreased from 2018 to 2022 among health workers (from 28.8% to 21.8%) and other essential workers (from 24.9% to 20.6%); however, overall, 78.2% of health workers in 2022 agreed or strongly agreed that they trusted management. Feeling that workplace conditions support productivity declined from 2018 to 2022 among health workers (from 30.4% to 16.2%) and other essential workers (from 19.0% to 12.8%). Overall, 81.5% of health workers agreed or strongly agreed that workplace conditions supported productivity. From 2018 to 2022, a higher percentage of health workers and other essential workers reported that there were often not enough staff members (from 25.7% to 32.0% and from 30.1% to 37.9%, respectively). Finally, presenteeism rates among health workers in 2022 (27.9%) were lower than rates in other essential workers (43.2%) and all other workers (37.4%).

Among health workers who reported being harassed, the odds of reporting anxiety, depression, and burnout were 5.01, 3.38, 5.83 times, respectively, those among health workers who were not harassed (Table 3). Compared with health workers who reported a poor psychosocial safety climate, the odds of reporting burnout were 0.35 and 0.24 times those among health workers who reported moderate and good psychosocial safety climates, respectively. Among health workers who reported that they trusted management and whose supervisors provided help, the odds of reporting burnout were 0.40 and 0.26 times, respectively, those among health workers who reported that they did not trust management or whose supervisors did not provide help. Health workers who took part in decision-making had 0.56 times the odds of reporting depression symptoms compared with health workers who reported they did not. Health workers who reported that there were not enough staff members had 1.91 times the odds of reporting symptoms of anxiety and 2.73 times the odds of reporting burnout compared with those who did not report staffing shortages. Health workers who reported having enough time to complete work had 0.33 times the odds of reporting burnout compared with health workers who did not. Finally, health workers who reported that conditions at work support productivity had 0.38 times the odds of reporting burnout compared with those who did not.

**TABLE 3:** Anxiety symptoms, depression symptoms, and burnout* of health workers (N = 325), by working conditions— General Social Survey Quality of Worklife Module, United States, 2022

Working conditions (no. with information)	Anxiety symptoms				Depression symptoms				Burnout			
	OR (95% CI)	%	Chi-square	p-value	OR (95% CI)	%	Chi-square	p-value	OR (95% CI)	%	Chi-square	p-value
**Harassment at work (313)^†^**												
No (271)	1 (—)	52.8	16.77	<0.01	1 (—)	30.6	12.83	<0.01	1 (—)	41.7	22.94	<0.01
Yes (42)	5.01 (2.45–10.26)	84.9			3.38 (1.53–7.47)	59.8			5.83 (2.56–13.27)	80.6		
**Psychosocial safety climate (310)^§^**												
Poor (35)	1 (—)	65.2	1.13	0.57	1 (—)	53.1	5.04	0.08^¶^	1 (—)	76.3	10.85	<0.01
Moderate (62)	0.74 (0.25–2.20)	58.2			0.34 (0.13–0.85)	27.6			0.35 (0.13–0.97)	53.3		
Good (213)	0.64 (0.28–1.49)	54.6			0.42 (0.18–0.97)	32.1			0.24 (0.09–0.61)	43.3		
Trust management (310)**												
Disagree (61)^††^	1 (—)	59.0	0.20	0.66	1 (—)	42.7	2.67	0.10	1 (—)	64.7	10.02	<0.01
Agree (249)^§§^	0.88 (0.41–1.88)	55.9			0.62 (0.32–1.21)	31.5			0.40 (0.19–0.86)	42.4		
**Supervisor helps (308)^¶¶^**												
Not true (50)***	1 (—)	55.2	0.06	0.80	1 (—)	40.0	0.85	0.36	1 (—)	73.3	16.47	<0.01
True (258)^†††^	1.08 (0.47–2.49)	57.2			0.74 (0.35–1.56)	33.0			0.26 (0.11–0.62)	41.8		
**Takes part in decisions (312)^§§§^**												
Never/Rarely (73)	1 (—)	60.4	0.30	0.58	1 (—)	45.3	4.06	0.04^¶^	1 (—)	38.7	2.59	0.11
Sometimes/Often (239)	0.86 (0.43–1.69)	56.7			0.56 (0.28–1.14)	31.8			1.57 (0.74–3.33)	49.8		
**Not enough staff (310)^¶¶¶^**												
Never/Rarely (98)	1 (—)	45.3	6.70	0.01	1 (—)	36.0	0.07	0.79	1 (—)	30.9	15.41	<0.01
Sometimes/Often (212)	1.91 (1.02–3.58)	61.3			0.93 (0.53–1.64)	34.3			2.73 (1.31–5.67)	54.9		
Time to get job done (312)****												
Not true (57)***	1 (—)	63.0	0.73	0.39	1 (—)	31.9	0.13	0.71	1 (—)	69.9	10.82	<0.01
True (255)^†††^	0.75 (0.35–1.59)	56.0			1.14 (0.63–2.07)	34.7			0.33 (0.16–0.66)	43.1		
**Conditions support productivity (312)^††††^**												
Disagree (58)^††^	1 (—)	61.4	0.48	0.49	1 (—)	50.0	6.31	0.01	1 (—)	66.7	9.62	<0.01
Agree (254)^§§^	0.80 (0.37–1.75)	56.1			0.45 (0.22–0.95)	31.2			0.38 (0.18–0.80)	43.0		

**Abbreviations**: GSS = General Social Survey; OR = odds ratio.

* All analyses used survey weights provided by GSS. Burnout dichotomized where never, rarely, and sometimes = 0 and often and very often = 1.

^†^ “In the last 12 months, were you threatened or harassed in any other way by anyone while you were on the job?” (GSS variable name: wkharoth).

^§^ Composite of GSS variables psysamephys (“Senior management considers psychological health to be as important as productivity”), strmgtsup (“Senior management show support for stress prevention through involvement and commitment”), and allorglevel (“In my organization, the prevention of stress involves all levels of the organization”). Response options: strongly disagree (1), disagree (2), neither agree nor disagree (3), agree (4), and strongly agree (5). Items were summed and scores <6 were coded “poor,” 6–8 were coded “moderate,” and ≥9 were coded “good.”

^¶^ p-values were estimated based on the chi-square of the model. Wald 95% CIs were estimated for the ORs.

** “I trust the management at the place where I work” (GSS variable name: trustman).

^††^ Strongly disagree and Disagree collapsed to create Disagree.

^§§^ Agree and Strongly agree collapsed to create Agree.

^¶¶^ “My supervisor is helpful to me in getting the job done” (GSS variable name: suphelp).

*** Not at all true and Not too true collapsed to create Not true.

^†††^ Somewhat true and Very true collapsed to create True.

^§§§^ “In your job, how often do you take part with others in making decisions that affect you?” (GSS variable name: wkdecide).

^¶¶¶^ “How often are there not enough people or staff to get all the work done?” (GSS variable name: toofewwk).

**** “I have enough time to get the job done” (GSS variable name: wrktime).

^††††^ “Conditions on my job allow me to be about as productive as I could be” (GSS variable name: prodctiv).

## Discussion

This study provides evidence that during the COVID-19 pandemic, U.S. health workers experienced larger declines in a range of mental health outcomes than did essential and other workers, with the exception of general happiness, which was lower in essential workers. These data support the imperative for action to create a system in which health workers can thrive, as described in the U.S. Surgeon General’s 2022 report “Addressing Health Worker Burnout,” (*8*) which notes that distressing work environments contributed to a record high number of health workers quitting their jobs. A population-based cross-sectional study in Norway in early 2020, at the beginning of the pandemic, reported lower levels of anxiety and depression among health care workers compared with other workers (*13*). In contrast, the current report finds that U.S. health workers reported a larger increase in number of days of poor mental health and burnout in 2022 compared with 2018 than did other workers, with nearly one half (46%) reporting burnout in 2022. U.S. health workers were also more likely than were other workers to report negative changes in working conditions during that time. In 2022, the prevalence of reported health worker harassment more than doubled, and the very likely intention to find another job increased by almost 50%. Negative working conditions are associated with higher prevalences of depressive symptoms (*1*,*2*), self-rated poor health (*14*), and turnover intention (*8*). Accordingly, the American Public Health Association^†††^ and the International Labour Organization promote decent work^§§§^ (e.g., work that provides security and social protection; a fair income; and opportunities for growth, development, and productivity) as a public health goal fundamental for protecting workers.

This report identifies modifiable working conditions that contributed to poorer mental health among health workers and suggests preventive actions for employers. Previous research found job stress interventions that changed aspects of the organization (e.g., increased manager social support) were more effective than were secondary (e.g., screening for stressors) or tertiary (e.g., individual stress management) (*15*) interventions. A recent review of management interventions suggests that training managers on mental health awareness and ways to support workers and improve safety culture shows promise for reducing worker stress and improving well-being (*16*). Working conditions that support productivity and foster trust in management might be more readily addressed than providing sufficient staffing, which can be challenging in resource-constrained settings. More positive psychosocial safety climates, which include management prioritization of psychological health and stress prevention, were associated with lower burnout symptoms among health workers in this study. Previous research has demonstrated the link between psychosocial safety climate and reduced exhaustion, improved worker well-being, and improved engagement (*17*). Organizational policies and practices can be modified to improve security and reduce threats of violence.^¶¶¶^ The International Organization for Standardization provides guidelines for managing psychosocial risks in the workplace to promote worker safety and health.**** Employers can also make changes that increase participation in decision-making and reduce workloads.^††††^ Evidence suggests that attention to such protective aspects of work could reduce the number of days of poor mental health and prevalences of burnout and turnover intention (*18*). Recent reviews note the limited number of organizational intervention studies addressing health worker mental health (*16*,*19*), reinforcing the need for researchers to join health employers, government, labor, and professional organizations in implementing effective organizational interventions and documenting their impact.

CDC’s National Institute for Occupational Safety and Health (NIOSH) has implemented efforts to promote the mental health and well-being of health workers. One is a national social marketing campaign, Impact Wellbeing, which emphasizes primary prevention strategies such as worker participation in decision-making, supportive supervision, and increasing psychological safety for help-seeking (*20*). NIOSH has also developed burnout prevention training for supervisors of public health workers.^§§§§^ Through these efforts, as noted in the Surgeon General’s report (*8*), the emphasis is on improving the work environment to support mental health, rather than asking workers to be more resilient or to fix problems themselves.

### Limitations

The findings in this report are subject to at least six limitations. First, the data are cross-sectional; causation cannot be inferred, and alternative explanations for the findings are possible. Second, these data are self-reported and subject to biases associated with recall and social desirability that could affect participant response. Third, because of administration during the pandemic, the 2022 GSS used mixed methods, including face-to-face and telephone interviews, and online administration; the 2018 survey was conducted using only face-to-face interviews. Use of these different methods might have influenced response rates and self-reporting of symptoms. Fourth, data were weighted to be nationally demographically representative, but were not adjusted for industry, occupation, and work setting. Fifth, a relatively small number of health workers were included in the 2022 sample. The fourth and fifth limitations might limit generalizability. Finally, measures of symptoms for anxiety and depression were not available in 2018, which precludes prepandemic comparisons.

### Implications for Public Health Practice

Health workers continued to face a mental health crisis in 2022. Improving management and supervisory practices might reduce symptoms of anxiety, depression, and burnout. Protecting and promoting health worker mental health has important implications for the nation’s health system and public health. Health employers, managers, and supervisors are encouraged to implement the guidance offered by the Surgeon General (*8*) and use CDC resources (*20*) to include workers in decision-making, provide help and resources that enable workers to be productive and build trust, and adopt policies to support a psychologically safe workplace.

## Footnotes

* Health occupations include direct patient-care workers such as nurses, physicians, emergency medical services clinicians, mental health workers, and long-term care workers, and those not engaged in direct patient care such as those in other support roles and public health workers. https://bhw.hrsa.gov/sites/default/files/bureau-health-workforce/about-us/hhs-health-workforce-strategic-plan-2021.pdf

† Working conditions include aspects of work design, the organization, and management of work, including but not limited to employment arrangements, organizational factors, job and task design, and social interactions.

§
https://www.osha.gov/news/newsreleases/national/02172022#:~:text

¶
https://gss.norc.org/Pages/quality-of-worklife.aspx

** Methods for GSS/QWL are described online (https://gss.norc.org/Get-Documentation). GSS is conducted in even-numbered years, and participants are recruited from nationally representative survey panels. The 2018 survey was administered via personal interview. The 2022 survey was administered via both personal and telephone interviews, as well as through a web-based questionnaire. Administration of GSS/QWL is covered by the National Opinion Research Center Institutional Review Board (Federal Wide Assurance number: FWA00000142). Data from GSS/QWL survey are publicly available. Nonresponse weights were used in the analyses.

†† Worker classifications were adapted from categories and industries defined by the Advisory Committee on Immunization Practices (https://www.cdc.gov/vaccines/covid-19/categories-essential-workers.html). North American Industry Classification System codes, published by CDC, were cross-referenced with industry codes for respondents’ employment provided in GSS. Health workers include those in the health occupations described above (direct care roles were not differentiated in these analyses); other essential workers include frontline, nonhealth workers; “all other workers” include all remaining workers.

§§ Psychosocial safety climate is shared perceptions within an organization about policies, practices, and procedures, that protect worker psychological health and safety. Items include “Senior management considers psychological health to be as important as productivity,” “Senior management show support for stress prevention through involvement and commitment,” and “In my organization, the prevention of stress involves all levels of the organization.” Responses (strongly disagree [1], disagree [2], neither agree nor disagree [3], agree [4], and strongly agree [5]) were summed. Scores <6 were coded “poor,” 6–8 were coded “moderate,” and ≥9 were coded “good.”

¶¶ 45 C.F.R. part 46, 21 C.F.R. part 56; 42 U.S.C. Sect.241(d); 5 U.S.C. Sect. 552a; 44 U.S.C. Sect. 3501 et seq.

*** Control variables were not used in these analyses because the weighting and complex sampling design of GSS/QWL accurately approximates the U.S. population. Moreover, using covariates to make groups statistically equivalent when known demographic differences exist might serve to diminish meaningful differences.

†††
https://www.apha.org/Policies-and-Advocacy/Public-Health-Policy-Statements/Policy-Database/2023/01/18/Decent-Work-for-All#:~:text

§§§
https://www.ilo.org/global/topics/decent-work/lang--en/index.htm

¶¶¶
https://www.osha.gov/sites/default/files/publications/osha3148.pdf

****
https://www.iso.org/standard/64283.html

††††
https://www.cdc.gov/niosh/twh/fundamentals.html

§§§§
https://www.cdc.gov/niosh/learning/publichealthburnoutprevention/default.html
